# Nonhormonal Pharmacotherapies for the Treatment of Postmenopausal Vasomotor Symptoms

**DOI:** 10.7759/cureus.52467

**Published:** 2024-01-17

**Authors:** Taylor Witten, Julia Staszkiewicz, Logan Gold, Mallory A Granier, Rachel J Klapper, Gabriel Lavespere, Bradley Dorius, Varsha Allampalli, Shahab Ahmadzadeh, Sahar Shekoohi, Alan D Kaye, Giustino Varrassi

**Affiliations:** 1 School of Medicine, Louisiana State University Health Sciences Center, Shreveport, USA; 2 School of Medicine, Louisiana State University Health New Orleans, New Orleans, USA; 3 Department of Radiology, Louisiana State University Health Sciences Center, Shreveport, USA; 4 Department of Anesthesiology, Louisiana State University Health Sciences Center, Shreveport, USA; 5 Department of Pain Medicine, Paolo Procacci Foundation, Rome, ITA

**Keywords:** snris, ssris, non-hormonal therapy, hormonal therapy, menopause, vasomotor symptoms

## Abstract

An average of 60-80% of all menopausal women experience bothersome vasomotor symptoms (VMSs), such as flushing and sweating, within the first seven years of onset. However, despite increasing prevalence, these hot flashes remain hard to treat and have a negative effect on the quality of life. Though hormone replacement therapy is commonly utilized as a standard treatment for VMSs, this therapy is not recommended for all women. Specifically, the oral form of hormone replacement therapy is associated with several contraindications, including a history of thromboembolic disease, migraine headache with aura, liver failure, heart disease, and hormone-dependent cancers. For women with these medical conditions, current literature indicates that nonhormonal therapies such as selective serotonin reuptake inhibitors (SSRIs) and serotonin-norepinephrine reuptake inhibitors (SNRIs) are suitable alternatives to reduce the frequency and intensity of VMSs. Currently, the only SSRI that is FDA-approved for the treatment of VMSs is paroxetine, but studies show that fluoxetine, citalopram, escitalopram, and sertraline are also proven to provide similar benefits. Similarly, the SNRI venlafaxine has also been well tolerated and has been shown to reduce the frequency and severity of hot flashes. The present investigation reviews the physiology of VMSs and examines the evidence for the use of nonhormonal pharmacologic therapies as treatment for women experiencing hot flashes. These interventions should be considered whenever hormone replacement therapy is contraindicated, with therapy individualized based on the severity of symptoms.

## Introduction and background

When women hear the word “menopause,” often what comes to mind are distressing vasomotor symptoms (VMSs). Despite it being only one of many features that can affect a woman experiencing menopause, the VMS is most associated with a debilitating impact on the quality of life. Based on the Study of Women’s Health Across the Nation (SWAN), an average of 60-80% of all menopausal women experience typical VMSs, such as hot flashes and night sweats [1]. This high prevalence of VMSs among menopausal women makes symptoms a common complaint. In this regard, studies have demonstrated a declining level of estrogen associated with aging, whereby ovaries shrink with associated dysregulation in the thermoregulatory system. Because of this imbalance, minute increases in core body temperature, as little as 0.8, can trigger vasodilation and sweating [2]. In addition, these episodes of VMSs can also be accompanied by agitation, anxiety, and depression. This culmination of symptoms has been shown to negatively impact the quality of life, affecting sleep quality, mood, relationships, and productivity [3]. Untreated, the VMS not only impacts a woman's quality of life at home and with family, but these symptoms also increase both the utilization of healthcare resources and costs, demonstrating the importance of effective treatment. In this regard, a study evaluating health insurance claims among women with untreated VMS revealed an average direct cost difference of $1,346 between affected women and those without VMS, further illustrating a significant financial burden without treatment [4-6]. Hormone replacement therapy is the most effective therapy for menopausal VMS, but its increased risk of estrogen-dependent pathologies warrants further investigation of other medications that are proven useful for this purpose [1-3]. The present investigation reviews the physiology of VMSs and examines the evidence for the use of nonhormonal pharmacologic therapies as treatment for women experiencing hot flashes.

## Review

Methods

We searched vasomotor symptoms, menopause, hormonal therapy, nonhormonal therapy, SSRIs, and SNRIs on PubMed, Google Scholar, MEDLINE, and ScienceDirect sources. Sources were accessed between September 2023 and December 2023.

Definition and epidemiology of VMSs

As a woman transitions out of her reproductive years and enters menopause, a common complaint is the onset of the VMS. Often referred to as "hot flashes," the VMS is characterized by episodes of overwhelming heat sweating and flushing of the skin of the upper back, chest, head, and neck [1]. The episodes can recur countless times during the day and night, lasting from one to five minutes and followed by chills [5]. Other symptoms that often accompany hot flashes include irritability, anxiety, depression, difficulty sleeping, concentrating, and short-term memory. The symptoms have been reported by women as early as several years before the onset of their last menstrual cycles, called perimenopause or menopausal transitions, and as late as into their 70s, presumably long after the transition from the reproductive to the menopausal stage [1]. Many treatment protocols, while hinging on a woman's presence or absence of a uterus, have been found to have greater benefit and efficacy in women under 60 years old [1]. 

The SWAN was conducted to assess the risk factors and prevalence of the VMS in menopausal women and showed that around 60-80% of women experience the VMS at some point during menopause, making this a significant medical issue for physicians to recognize and treat [1]. When looking at the highest reported prevalence of symptoms, African-American women were the most likely to report the VMS. The SWAN study found that Chinese and Japanese women were the least likely racial groups to report the VMS and were least likely to label the symptoms as bothersome. One of the key risk factors identified in the SWAN study was obesity, with women with a higher body mass index (BMI) reporting more bothersome and more frequent VMS than those women with a lower BMI. A cross-sectional study that directly looked at obesity and its relationship with the VMS showed that weight loss may help manage hot flashes, with studies finding a positive correlation between increased abdominal circumference and severity of the VMS [7]. Another risk factor identified was smoking, with smokers and women experiencing secondhand smoke having a 60% increased chance of experiencing reportable symptoms. Preexisting anxiety and depression were also shown to have an increased risk of more frequent VMS and reported a higher severity of symptoms [8]. Other risk factors include a history of child abuse, low socioeconomic status, and lower educational level. However, these risk factors have been found to have confounding variables with mental illness, smoking, and obesity and, therefore, cannot be directly associated with the VMS [1].

Studies have also focused on how the VMS influences health status and the number of resources used to treat women. Links have also been found between the VMS and perceived sleep disturbances, higher levels of depression, and a potential transient decrease in cognition. It has also been found that increased severity of the VMS resulted in more frequent medical visits, with the cost of healthcare increased by 123% for women experiencing mild symptoms, 224% for moderate, and 274% for severe symptoms [5]. VMSs in postmenopausal women have also been linked to poor endothelial function, aortic calcification, and atherosclerosis [1].

Physiology of VMSs

VMSs are hypothesized to be caused by a decrease in the range of the thermoneutral zone, in which the core body temperature is kept without triggering compensatory mechanisms like sweating or shivering. This means that more minor changes in core temperature can trigger peripheral vasodilation and sweating, causing a “hot flash.” As women enter menopause, the menstrual cycle stops, and the decrease in circulating estradiol and progesterone coincides with the incidence of the VMS, leading scientists to believe that VMSs are a direct effect of declining gonadal hormones [9]. In the SWAN study, higher levels of follicle-stimulating hormone and lower levels of estradiol were linked to a greater likelihood of reporting VMS.

One hypothesis for the occurrence of the VMS about hormonal changes during menopause describes the function of estrogen in thermoregulation in the central nervous system. This hypothesis states that because throughout a woman's life, her brain has to adapt to fluctuating levels of hormones, including estrogen, the disruption in thermoregulation during menopause comes from the brain's inability to adjust to low estrogen levels. However, prepubertal children do not experience hot flashes, leading to the belief that while estrogen plays a role in the triggering of this thermoregulatory dysfunction, there have to be other components at play [10]. A key element of thermoregulation in the body is the hypothalamus, which expresses progesterone and estrogen receptors. With falling gonadal levels, the hypothalamus may experience changes in function, leading to downstream effects on serotonin and norepinephrine, other hormones involved in thermoregulation. Estrogen has also been shown to influence serotonin and norepinephrine synthesis, synaptic binding site density, and neurotransmitter reuptake and degradation. The disruption of norepinephrine and serotonin levels is hypothesized to be one method of thermoregulation disruption leading to the VMS, as clinically demonstrated with the efficacy of selective serotonin reuptake inhibitors (SSRIs) and serotonin-norepinephrine reuptake inhibitors (SNRIs) alleviating the VMS in postmenopausal women [9].

While the hypothalamus plays a part in thermoregulation, other studies that have focused on imaging the brain during vasomotor episodes showed activation of the insular and the anterior cingulate cortex, while sweating in nonmenopausal women showed activation of the anterior cingulate and superior frontal gyrus [10]. The sudden rush of heat that many women with hot flashes describe may be attributed to insular activity [10].

A separate study found that the brainstem activates before a hot flash, which may reflect the origin of the vasomotor event, followed by the activation of the insular cortex. Some hypothesize that other factors, such as genetics and obesity, may play a role in hot flashes' physiology or intensity. One theory is that adipose tissue acts as an insulator and prevents heat from dissipating during normal thermoregulatory processes, causing an increase in core body temperature and symptoms of a hot flash [7]. Another proposed hypothesis uses the above-described mechanism of estrogen involvement in the thermoregulatory process. It postulates that the increased adipose tissue in overweight women increases the amount of circulating estrogens and works to decrease the symptoms of hot flashes. This is, however, contradictory to studies that showed increased severity and rate of the VMS in overweight women [1]. Estrogen receptor polymorphisms and selected single-nucleotide polymorphisms have been studied to find a link between genetics and VMSs, with results showing a link between VMSs and sex hormone activity alterations related to polymorphisms [1].

Contraindications to hormonal therapy for the VMS

The transition to menopause is a distressing time for many women, serving as a constant reminder of their fleeting youth and fertility. While the VMS is only one constellation of menopausal symptoms, they can markedly decrease the quality of life. A recent European study that assessed this quality-of-life burden showed that the VMS of menopause is implicated in disordered sleeping, sexual dysfunction, and mental health decline, among others [11-13]. The gold standard treatment for symptomatically managing VMS is hormone therapy, which replenishes hormones that are no longer endogenously made by postmenopausal women [14]. As women approach menopause, ovaries shrink in size and gradually stop producing estrogen [15]. This lack of estrogen is responsible for VMSs as the thermoregulatory zone narrows, leading to transient flashes of heat and sweating. Low estrogen levels during the menopausal transition period also place women at higher risk for serious health concerns, including osteoporosis and cardiovascular disease [12]. Hormone therapy is indicated for the following menopausal issues: (i) treatment of VMSs of menopause, (ii) treatment of genitourinary syndrome of menopause, and (iii) prevention of osteoporosis [16].

Based on the American College of Obstetricians and Gynecologists (ACOG), hormone replacement options can be divided into estrogen-only and estrogen-plus progestin therapy, and they can be administered either systemically or locally. Estrogen-only therapy increases the lifetime risk for the development of endometrial cancer. Thus, it is not recommended for women without hysterectomy [17]. Estrogen plus progestin regimens are better treatment options for these patients, as progestin provides a certain level of endometrial protection. Combined hormone therapy with estrogen and progestin has been correlated with an enhanced risk of breast cancer and a reduced risk of colorectal cancer in various studies [15,18]. The choice to start hormone therapy for the treatment of VMSs of menopause should be a shared decision between the patient and the provider, considering the risks and benefits with careful consideration of the patient’s personal and family health histories.

While hormone therapy is effective in symptomatically managing the physical manifestations of menopause, many women are not candidates for treatment [6,12,14]. Contraindications to hormone therapy include previous hormone-dependent cancer, vaginal bleeding of unknown origin, acute liver disease/failure, history of deep vein thrombosis, migraine headache with aura, and history of heart disease. It should also be avoided in pregnant women or those who may become pregnant [6,12,13]. Even in the absence of contraindications, many women prefer nonhormonal therapies for VMS treatment. In recent years, there has been a growing research interest in nonhormonal medications for these patients, including SSRIs, SNRIs, selective estrogen modulators (SERMs), and anticonvulsants such as gabapentin [6,15].

Efficacy of treatment with SSRIs 

While not as effective as hormone replacement therapies, SSRIs are effective in reducing the frequency and intensity of hot flashes in menopausal women [6,12,15]. On the molecular level, estrogen helps modulate serotonergic signaling by increasing the density of serotonin receptors in certain tissues and directly increasing serum serotonin levels via tryptophan hydroxylase regulation [19]. Thus, as estrogen production declines during menopause, serotonin levels also drop. This hormonal interplay is likely implicated in not only the VMS that accompanies menopause but also mood symptoms such as depression and anxiety. This close physiological relationship between estrogen and serotonin is thought to influence various other bodily systems, including skeletal, vascular, and immune [19]. The only SSRI that is currently FDA-approved for VMS treatment is paroxetine, but studies show fluoxetine, citalopram, escitalopram, and sertraline are also proven to provide similar benefits [6]. A 2017 comprehensive multi-study analysis revealed that paroxetine, citalopram, and escitalopram are more helpful than the other SSRIs in decreasing the frequency and intensity of VMSs, reportedly by 10% to 64% [20].

General adverse effects of SSRIs include nausea, vomiting, diarrhea, headache, agitation, sexual dysfunction, and insomnia [21]. These mild adverse effects will resolve after the first few weeks of treatment [20,21]. The SSRIs’ favorable side effect profiles make them generally more tolerable than similar medications, such as SNRIs [19,20,21]. Except for paroxetine, which can behave as a dual SNRI at specific doses, SSRIs have little to no effect on other neurotransmitters [22]. Table 1 illustrates some drug-specific adverse effects as well as overall contraindications to the SSRI drug class. These drugs should not be taken in conjunction with any other medications that increase the availability of serotonin within the synaptic cleft, such as monoamine oxidase inhibitors (MAOIs). When taken together, these medications significantly increase the risk of developing serotonin syndrome, a potentially lethal condition characterized by high fever, agitation, altered mental status, tachycardia, tremors, and blood pressure alterations [21-23]. The FDA provided a black box warning for SSRIs in 2004 when it was found that children and young adults under age 25 who regularly take SSRIs are at increased risk for suicidal thoughts and behaviors. It is thus recommended that patients under age 25 should be regularly monitored for unusual changes in behavior that may indicate suicidal ideation [22]. Menopausal patients or those with known psychiatric conditions should also be closely monitored for depressive symptoms, as they are similarly at increased risk for suicidal ideation [22]. As with hormone replacement therapy, the decision to begin SSRI treatment for VMSs of menopause should be tailored to the individual by assessing risks and benefits in the context of the patient’s clinical picture.

**Table 1:** First- and Second-Line SSRIs Indicated for the Treatment of Menopausal Hot Flashes SSRIs: Selective serotonin reuptake inhibitors; MAOIs: monoamine oxidase inhibitors; VMS: vasomotor symptoms Sources [6,22,23]

SSRI	FDA-Approved for VMS?	Contraindications	Adverse Effects
Paroxetine	Yes	Concurrent use of MAOIs, linezolid (↑ risk serotonin syndrome) Pregnancy (teratogenic in 1^st^ trimester) Black Box Warning: Increased risk of suicidal thoughts and behaviors among children and adolescents. Must be closely observed for clinical worsening, suicidality, or unusual changes in behavior.	Nausea
Citalopram	No		Withdrawal, prolonged QT interval
Escitalopram			Withdrawal, nausea, weakness, drowsiness
Fluoxetine			Withdrawal
Sertraline			Nausea, sexual dysfunction

Efficacy of treatment with SNRIs

The decline in estrogen levels correlated with menopause is thought to dysregulate the serotonergic and noradrenergic activity of the thermoregulatory system, leading to VMSs like hot flashes, flushing, and sweating with core body temperature changes as small as 0.8± 0.99°C [24]. For this reason, SNRIs are proposed as an alternative to the accepted hormonal treatment for VMSs in menopausal women [24]. Venlafaxine, desvenlafaxine, and duloxetine are examples of drugs within this class and are widely prescribed to treat depression, anxiety, and chronic pain [25,26]. By blocking the neurotransmitters’ reuptake, they increase the availability of serotonin and norepinephrine in the synapse, and downstream effects, including serotonin receptor regulation, have been hypothesized [19].

In a 2013 study by Pinkerton et al., desvenlafaxine was shown to be a safe and well-tolerated alternative to hormonal treatment for VMSs, showing a significant decrease in the frequency and severity of hot flashes. The frequency of hot flashes was decreased by 60-65% with desvenlafaxine treatment, which falls within the reported range for the currently recommended low-dose hormonal therapy [24]. Venlafaxine has also been well tolerated and was shown to reduce hot flash frequency and severity and improve sleep quality in previous randomized controlled trials (RCTs) [27,28]. In a separate study, venlafaxine resulted in a 48% reduction of VMS frequency, while the traditional estradiol treatment resulted in a 53% reduction, and the placebo resulted in a 29% reduction [29]. This study concluded that estradiol and venlafaxine treatments have only modest differences in their efficacy in treating VMSs of menopause.

Venlafaxine treatment in women with a history of breast cancer and current VMSs showed a significant reduction in hot flashes in comparison with placebo in two RCTs in breast cancer survivors [30,31]. This improvement was seen as early as week four of treatment in 42% of cases [32]. Due to their proven safety and effectiveness, the American Cancer Society and the American Society of Clinical Oncology suggest SNRIs as the treatment of choice for VMSs in women with a history of breast cancer [33]. Since then, venlafaxine has become the most widely prescribed antidepressant for VMS relief.

Compared to placebo, desvenlafaxine was shown to cause significant increases in blood pressure, pulse rate, and lipid levels. Other adverse effects reported include xerostomia, nausea, constipation, diarrhea, fatigue, and drowsiness [24]. Loprinzi et al. showed that xerostomia, nausea, and constipation were also associated with venlafaxine treatment as well [31]. Rarely, suicidal thoughts have been established as an adverse effect of SNRIs in women with concurrent depression, as the dose required to achieve VMS relief is lower than what is needed to treat depressive symptoms [25,26]. Additionally, SNRIs can interfere with the cytochrome P450 2D6 (CYP2D6) enzyme required for the metabolism of tamoxifen, a breast cancer therapy. Among the SNRIs, venlafaxine and desvenlafaxine are mild inhibitors of CYP2D6 and safer to use with tamoxifen, while duloxetine is a moderate inhibitor [32,26]. Major contraindications to the use of serotonergic drugs include the concurrent use of a monoamine oxidase inhibitor or previous episodes of neuroleptic and/or serotonin syndrome. Also, these drugs should be used with caution in patients with known CYP2D6 polymorphisms, bipolar disorder, kidney/liver dysfunction, hyponatremia, or uncontrolled hypertension [25,26]. The North American Menopause Society (NAMS) also suggests caution with serotonergic drugs in patients affected by uncontrolled seizures or those currently using other SSRIs or SNRIs [26].

Despite their use in the treatment of VMSs of menopause, SNRIs also have the potential to worsen sexual function, as they can affect dyspareunia, orgasm potential, lubrication, and libido. Serotonergic drugs, specifically venlafaxine, used at higher doses for the treatment of depression, are also known to cause decreased sexual function. However, this seems to be a dose-dependent effect. The venlafaxine doses recommended for the treatment of VMSs are lower than those used in depression, and the few trials that have been done have shown limited evidence of sexual dysfunction [30,31]. An RCT of low-dose estradiol vs. venlafaxine for hot flashes showed no change in overall sexual function over eight weeks in either group compared to the placebo. However, a subtle increase in sexual desire was found in the estradiol group, while a decrease in orgasm and dyspareunia was found in the venlafaxine group [27]. Treatment decisions should, therefore, be individualized to consider the patient’s level of concerns for sexual function (Tables 2 and 3). 

**Table 2:** Comparative Studies of SNRIs vs. Alternatives SNRIs: Serotonin-norepinephrine reuptake inhibitors

Author (Year)	Groups Studied and Intervention	Results and Findings	Conclusions
Reed et al., 2014 [27]	An 8-week randomized controlled trial was done on 256 menopausal women experiencing hot flashes to compare oral estradiol and venlafaxine, as well as evaluate possible sexual function changes.	Overall sexual function was not significantly altered with venlafaxine or estradiol compared to placebo. However, estradiol treatment did show a slight increase in libido, and venlafaxine showed slight decreases in dyspareunia as well as the ability to orgasm.	Venlafaxine is an effective alternative to estradiol for the treatment of hot flashes in menopausal women, without significant effects on sexual function. Treatment should be personalized based on potential side effects and desire for sexual function.
Joffe et al., 2014 [29]	339 perimenopausal and postmenopausal women experiencing ≥ 2 bothersome vasomotor symptoms daily were placed in an 8-week randomized clinical trial to assess the efficacy and safety of low-dose venlafaxine, compared to estradiol.	Venlafaxine treatment was well tolerated and showed a significant reduction in the frequency of vasomotor symptoms.	Low-dose venlafaxine is an effective and safe alternative to estradiol for the treatment of vasomotor symptoms associated with menopause. The efficacy of venlafaxine was just slightly inferior to estradiol.
Ensrud et al., 2015 [28]	339 perimenopausal and postmenopausal women experiencing ≥ 2 hot flashes daily were placed in an 8-week double-blind, randomized trial to determine the effects of low-dose estradiol and venlafaxine on sleep quality and insomnia.	Sleep quality, which was determined by self-reported subjective sleep measures, was improved slightly with both venlafaxine and estradiol treatment compared to placebo.	It is clinically relevant to conclude that venlafaxine and estradiol treatment have similarly modest effects on the improvement of sleep quality and insomnia, which are common complaints associated with vasomotor symptoms.
Boekhout et al., 2011 [32]	102 women with a history of breast cancer treatment and hot flashes were placed in a double-blind, placebo-controlled trial in which they received clonidine, venlafaxine, or placebo for 12 weeks.	Venlafaxine treatment was associated with a fast-acting reduction in hot flashes. Clonidine, however, at the end of the 12 weeks showed a more efficacious reduction in hot flashes.	Venlafaxine and clonidine are both effective alternatives to hormonal therapy in the reduction of hot flashes in patients with a history of breast cancer. Venlafaxine is associated with more immediate relief of symptoms.

**Table 3:** Clinical Efficacy and Safety of SNRIs NAMS: The North American Menopause Society; SNRIs: serotonin-norepinephrine reuptake inhibitors

Author (Year)	Group Studied and Intervention	Results and Findings	Conclusions
Pinkerton et al., 2013 [24]	365 postmenopausal women experiencing ≥ 50 moderate to severe hot flashes weekly were placed into a 12-week randomized, double-blind placebo-controlled efficacy trial of desvenlafaxine for the treatment of vasomotor symptoms.	Desvenlafaxine showed a significant reduction in hot flash frequency and severity, compared to the placebo.	Desvenlafaxine showed rapid symptom reduction and is a safe and an effective alternative to hormone replacement therapy for the treatment of hot flashes.
Carpenter et al., 2007 [30]	87 breast cancer survivors with vasomotor symptoms were placed into a randomized, double-blind, placebo-controlled trial of venlafaxine (at low vs. high dose) to determine symptom reduction.	Venlafaxine resulted in a modest reduction in hot flashes at low doses and even better improvement with higher doses. With the significant improvement of hot flashes, also came improvements in feelings of fatigue, sleep, and quality of life. Though side effects were mild, many discontinued long-term use of venlafaxine.	Venlafaxine is an effective and fast-acting drug that reduces hot flashes in breast cancer survivors with few side effects. However, it may not be well-tolerated for long-term use and, unfortunately, requires a ≥ 50% reduction in hot flashes to improve sleep and quality of life.
Loprinzi et al., 2020 [31]	221 women with a history of breast cancer (and therefore contraindication to hormonal treatment) or reluctance due to the risk of cancer were placed in a double-blind placebo-controlled randomized trial of venlafaxine treatment at various doses.	The participants filled out a daily questionnaire that considered hot flash frequency and severity, as well as the side effects experienced. After only 4 weeks of treatment, venlafaxine showed significant reductions in hot flashes, especially at higher doses. However, adverse effects including xerostomia, anorexia, constipation, and nausea were seen more frequently at higher doses as well.	Venlafaxine is an effective nonhormonal alternative for the treatment of vasomotor symptoms in patients with contraindications or fear of the risks. However, the dosage prescribed must balance the efficacy of the treatment with the possible side effects.
NAMS, 2015 [26]	The North American Menopause Society reviews and compares potential nonhormonal therapy options for vasomotor symptom management in those who cannot or are reluctant to take hormonal therapy.	Through literature review and expert consensus, NAMS found SNRIs to be a potential effective alternative to hormone replacement therapy for the treatment of hot flashes.	SNRIs are an effective nonhormonal option for the treatment of vasomotor symptoms when hormonal therapy is unwanted or contraindicated.

Discussion 

Women have reported VMSs beginning as early as the years before cessation of menstrual cycles to as late as their seventh decade of life. An increase in the reported severity of these symptoms has been found to result in more frequent medical visits and higher healthcare expenses [5]. The decline in estrogen levels correlated with menopause is thought to dysregulate the serotonergic and noradrenergic activity of the thermoregulatory system in the hypothalamus. A smaller change in core body temperature can result in peripheral vasodilation, sweating, and a hot flash [24,25]. Because of their ability to increase the availability of serotonin and norepinephrine, SSRIs and SNRIs were proposed as an alternative to the accepted hormone replacement therapy to treat VMSs in postmenopausal women.

Currently, the standard treatment for VMS relief is hormone replacement therapy, which replenishes the diminished estrogen that is made by the atrophic ovaries after menopause [14]. Contraindications to hormone replacement therapy include previous hormone-dependent cancer, idiopathic vaginal bleeding, liver or heart disease, history of thrombosis, or migraine headache with aura. Therefore, many women are not possible candidates for hormonal treatment.

This highlights the need for nonhormonal medication options for these patients, including SSRIs and SNRIs. Multiple investigations have demonstrated these two alternate therapies have shown significant reductions in hot flash frequency and intensity and might be better options for women with contraindications to hormonal therapy [6,12,15,27,28]. The only SSRI that is currently FDA-approved for the treatment of VMSs is paroxetine, but others, including fluoxetine, citalopram, escitalopram, and sertraline, have shown similar efficacy [6]. The adverse effects seen with SSRIs include nausea, vomiting, diarrhea, headache, sexual dysfunction, and insomnia [21 ]. However, these are often mild and resolve after the first week of treatment [20]. The tolerable side effect profiles and effectiveness at reducing VMSs make SSRIs a possible class of alternatives to hormonal therapy. Contraindications to SSRIs include the concomitant use of a monoamine oxidase inhibitor.

## Conclusions

There exists a stigma that nonhormonal pharmacotherapy options are suboptimal to hormone replacement therapy for treating symptoms of menopause and that antidepressants cause sexual dysfunction and other unappealing side effects. Among the SNRIs, desvenlafaxine and venlafaxine have been shown to be safe, well-tolerated, efficacious, nonhormonal treatment options for the relief of hot flashes. Venlafaxine has shown only a modestly inferior efficacy to estradiol treatment. Because of their safety and efficacy, the American Cancer Society and the American Society of Clinical Oncology recommend SNRIs as the treatment for VMSs in women with a history of breast cancer. Because these treatment options come with various contraindications and potential side effects, the choice of therapy should be a shared decision between the patient and the provider, taking into consideration the patient’s concerns and their personal and family medical histories. Given that nonhormonal pharmacotherapy options are comparable in treating VMSs of menopause, without the increased risks of breast cancer and with minimal evidence of unappealing side effects, there exists an increased role for these drugs in women's healthcare.
